# Transcriptome-Wide 5-Methylcytosine Profiling of lncRNAs in the Mouse Cerebral Ischemia Model

**DOI:** 10.3390/ph17030384

**Published:** 2024-03-18

**Authors:** Chao Zhang, Junpeng Gao, Dan Xiong, Yan Zhao

**Affiliations:** 1 Emergency Center, Zhongnan Hospital of Wuhan University, Wuhan 430071, China; 2021283030120@whu.edu.cn (C.Z.); gaojunpeng@pku.edu.cn (J.G.); danxiong@whu.edu.cn (D.X.); 2 Hubei Clinical Research Center for Emergency and Resuscitation, Zhongnan Hospital of Wuhan University, Wuhan 430071, China

**Keywords:** lncRNAs, 5-methylcytosine, stroke, middle cerebral artery occlusion, MeRIP-seq

## Abstract

An increasing body of research has demonstrated the significant role of long non-coding RNAs (lncRNAs) in the pathogenesis of stroke. They can actively contribute to the disease’s progression either by directly participating in its pathogenesis or by acting as mediators through competing endogenous RNA (ceRNA) mechanisms. Concurrently, epigenetics plays a pivotal role in the pathological mechanisms underlying stroke. Epigenetic factors serve as valuable markers for disease progression, diagnostic biomarkers, and novel therapeutic targets. One of the most prevalent epigenetic modifications is 5-methylcytosine (m^5^C). However, the specific profiles of 5-methylcytosine in lncRNAs associated with stroke remain to be solved. Within the scope of this research, we performed a thorough transcriptome-wide analysis of m^5^C methylation within lncRNAs by methylated RNA immunoprecipitation sequencing (MeRIP-Seq), within a mouse stroke model induced by middle cerebral artery occlusion. Our findings reveal substantial disparities in both the quantity and distribution of m^5^C within the mouse stroke model compared to normal mice. This suggests a potential linkage between stroke and lncRNA m^5^C modifications, offering valuable insights into the mechanisms of stroke pathogenesis and the development of new drug targets.

## 1. Introduction

Stroke stands as a highly morbid, lethal, and disabling ailment on a global scale [1,2]. The worldwide prevalence of stroke is estimated to be approximately 2.7%. Statistics indicate that around 7.6 million individuals aged 20 and above in the United States have encountered strokes, with approximately 795,000 individuals experiencing new or recurrent strokes annually. Of all stroke cases, 87% are attributed to ischemic strokes [3]. Ischemic strokes occur when blood vessels become obstructed, resulting in reduced blood flow, cellular death, disruption of cell membranes, leakage of cellular contents, and a loss of neuronal function [4], Moreover, research has demonstrated that oxidative stress, toxicity mediated by free radicals, complement activation, disruption of the blood-brain barrier, and inflammation also play pivotal roles in the pathophysiological processes underlying stroke [5,6,7,8,9].

Epigenetics refers to the process of regulating gene expression without altering the underlying DNA sequence. These regulatory mechanisms encompass DNA methylation or hydroxymethylation, histone modification, chromatin remodeling, and RNA methylation [10]. Epigenetic mechanisms play an important role in regulating various physiological processes within organisms, including cell proliferation and differentiation. Notably, recent research has uncovered the significance of epigenetics in the regulation of gene expression related to stroke in humans [11]. There is accumulating evidence supporting the role of epigenetic changes in the incidence of cardiovascular disease in both human subjects and animal models, which subsequently heightens susceptibility to stroke [12]. Epigenetic regulators represent a novel class of multifaceted drug targets capable of finely tuning the expression of thousands of genes, thereby reshaping the injured brain [13]. Studies have demonstrated that key epigenetic regulators can be targeted using enzyme inhibitors, small molecules, miRNA mimics, and antagonists. These approaches not only hold the potential to prevent and ameliorate damage caused by ischemic injury and oxidative stress following a stroke but also facilitate post-stroke recovery by inducing neurogenesis, angiogenesis, and synaptic plasticity [14,15,16,17].

RNA methylation, as a post-transcriptional modification, may affect gene expression by affecting splicing, RNA metabolism, stability, and translation [18]. Among the post-transcriptional modifications of RNA, 2/3 were methylated, including m^1^A, m^5^C, and m^7^G. 5-methylcytosine (m^5^C) of RNA refers to methylation on the fifth carbon atom of RNA cytosine [19]. M^5^C is a conservative and ubiquitous modification in RNA which is most abundant in eukaryotic tRNA and rRNA [20]. M^5^C modification is mainly mediated by methyltransferase (writer), demethylase (eraser), and binding protein (reader). By interacting with a variety of “writers”, “erasers” and “readers” proteins, m^5^C modification extensively affects gene expression. Previous studies, which detected about 7541 m^5^C sites in mouse embryonic stem cells and 2075 m^5^C sites in mouse brains, concluded that m^5^C modifications were predominantly present in the coding region and were near the translation initiation site and in poly(A) [21]. And, the role of m^5^C in non-coding RNA was complex and can protect tRNA from endonucleic acid cleavage of angiopoietin [22]. M^5^C was also detected in small ncRNAs and lncRNAs [23,24]. M^5^C plays various important roles in lncRNAs. For example, the m^5^c demethylase TET2 protein binds to the promoter region of the oncogenic long non-coding RNA (lncRNA-ANRIL) and regulates the expression of ANRIL and its downstream genes, thereby reducing the risk of gastric cancer [25]. TET2 protein plays a tumor-suppressive role in prostate adenocarcinoma by enhancing immune cell infiltration [26]. Additionally, the m^5^c methyltransferase NSUN2 increases the stability of the tumor-associated lncRNA H19 through methylation in hepatocellular carcinoma. Many cancers exhibit abnormal overexpression and tumorigenic effects of H19 [27]. NSUN2 methylates lncRNA NR_033928, interacts with the IGF2BP3/HUR complex to upregulate the expression of glutaminase (GLS), promoting the stability of GLS mRNA and advancing gastric cancer [28]. The epigenetic characteristics of m^5^C-related lncRNAs also have prognostic significance [29]. Yuan H. et al. demonstrated significant differences in the expression of m^5^C-related lncRNAs between pancreatic cancer and normal tissues. They identified eight m^5^C-related lncRNAs and their clinical charts that can predict 3-year survival [30].

Ischemic injury is known to result in increased DNA methylation following the transcriptional repression of a substantial number of genes, ultimately contributing to exacerbated brain damage [31]. Notably, during this process, ischemia-induced production of reactive oxygen species and reactive nitrogen species (ROS/RNS) appears to influence CpG island methylation by converting 5-methylcytosine (5-mC) to 5-hydroxymethylcytosine (5-hmC). This conversion inhibits transcription and modulates methylation patterns by impeding the binding of DNA methyltransferase 1 (Dnmt1) and methyl-CpG-binding protein (MBP) to DNA [32]. The significance of DNA methylation extends to its correlations with stroke outcomes. Particularly, the methylation pattern of SLC6A4 in blood has been associated with the risk of stroke recurrence and prognosis [33]. Moreover, the biological age estimated through the level of m^5^C in circulation can provide a more accurate prediction of stroke risk, outcomes, and recurrence compared to chronological age alone [34,35]. Consequently, m^5^C modification emerges as a promising target and marker in the realm of stroke diagnosis, prognosis, and treatment.

Long non-coding RNAs (lncRNAs) are characterized as non-coding transcripts exceeding a length of 200 nucleotides. Due to their substantial fraction in the mammalian transcriptome, lncRNAs have garnered increasing attention [36]. In recent years, an expanding body of research has illuminated the widespread involvement of lncRNAs in the intricate regulation of gene expression. They assumed crucial functions in processes like cell growth, differentiation, programmed cell death, migration, invasion, and drug resistance [37]. Concurrently, lncRNAs have emerged as key players in the development and occurrence of stroke. Research has uncovered abnormal expression patterns of multiple lncRNAs following ischemic stroke [38]. Additionally, lncRNAs may contribute to the pathogenesis of ischemic stroke through various mechanisms, including calcium overload, oxidative stress, hypoxia, pressure signals, vascular pathological changes, neuronal necrosis, and inflammatory responses [39,40]. One particular lncRNA, maternally expressed gene 3 (Meg3), has shown significant expression in middle cerebral artery occlusion (MCAO) rats, and inhibiting Meg3 has been found to reduce OGD/R-induced apoptosis [41]. However, the precise role of m^5^C modification in ischemic stroke remains unclear. Given the challenges in obtaining human stroke samples, researchers commonly utilize the internationally recognized MCAO model to simulate cerebral ischemia. This model closely mimics the pathogenesis of human ischemic stroke and holds great significance for elucidating the pathogenesis and screening drugs for cerebral ischemia [42,43]. To explore epigenetic RNA modifications in MCAO and assess changes in neurological function following ischemic stroke, we conducted a transcriptome-wide analysis of the m^5^C methylation profile in MCAO mice by MeRIP-Seq.

## 2. Results

### 2.1. Increase inCerebral Infarction Area and Aggravation of Neurological Impairment in MCAO Group

**Figure 1:**
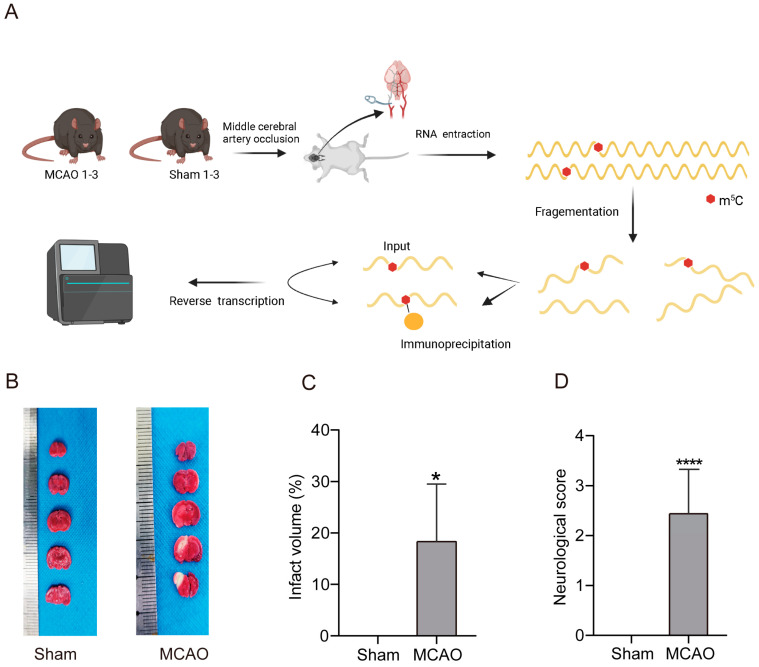
Constructed mouse model of MCAO. (**A**) Schematic diagram of experimental design. (**B**) TTC staining images of brains in two groups to detect infarcted area. The white area represented the infarcted area and the red area represented non-infarcted area. (**C**) The percentage of cerebral infarction volume in total brain volume in the MCAO group and the Sham group *(n* = 16, *p* < 0.05). (**D**) The neurological impairment score of mice in the MCAO group and the Sham group (*n* = 16, *p* < 0.0001). *: *p* < 0.05, ****: *p* < 0.0001.

To explore lncRNA m^5^C modifications in stroke, we firstly constructed mouse model of MCAO (Figure 1A). The results of 2,3,5-Triphenyltetrazolium chloride (TTC) staining revealed the presence of prominent white infarctions in the brain tissues of mice belonging to the middle cerebral artery occlusion (MCAO) group (Figure 1B). Subsequently, the infarct volumes for each experimental group were calculated and subjected to statistical analysis (Figure 1C). The analysis demonstrated that the percentage of infarct volume in the MCAO group was 18.33% ± 7.92%, which was a significant increase from the corresponding value in the Sham group. Furthermore, we assessed the neurological deficit using the Longa neurological score and found that mice in the Sham group displayed no signs of neurological impairment, yielding a Longa neurological score of 0. Conversely, the Longa neurological score in the MCAO group was 2.44 ± 0.22 (Figure 1D), indicating a more severe neurological deficit compared to the Sham group. These results showed that the construction of MCAO models was successful.

**Figure 2:**
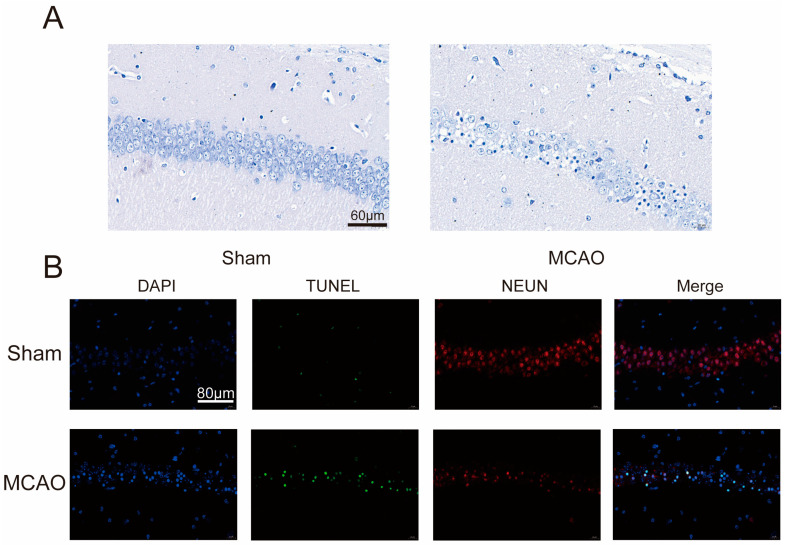
MCAO group mice exhibited clear neural damage. (**A**) Nissl staining of brain sections, under a biological microscope. Nissl bodies appear purple-blue, nuclei are blue, with a scale bar of 60 μm. (**B**) Representative images of brain sections co-stained with DAPI, TUNEL, and NEUN. Scale: 80 μm. Apoptotic cells on tissue slices exhibit green fluorescence and neuron appears as red fluorescence under a fluorescent microscope.

To explore the extent of neural damage in the MCAO group mice, we initially examined the survival status of neurons. As shown in Nissl staining (Figure 2A), neurons in the control group displayed clear neural structures with evenly distributed Nissl bodies, while neurons in the MCAO group exhibited significant disarray in their structures. Therefore, we proceeded to assess neuronal apoptosis further through TUNEL staining. Brain samples were subjected to immunofluorescent staining to detect co-expression of TUNEL and NEUN (Figure 2B). Following MCAO, there was a reduction in NEUN fluorescence intensity in the brain tissue, accompanied by an increase in TUNEL fluorescence intensity. These findings indicate a pronounced occurrence of neural apoptosis in the MCAO group mice.

### 2.2. General Characteristics of lncRNAs m^5^C Methylation in MCAO Model and Normal Mice

**Figure 3:**
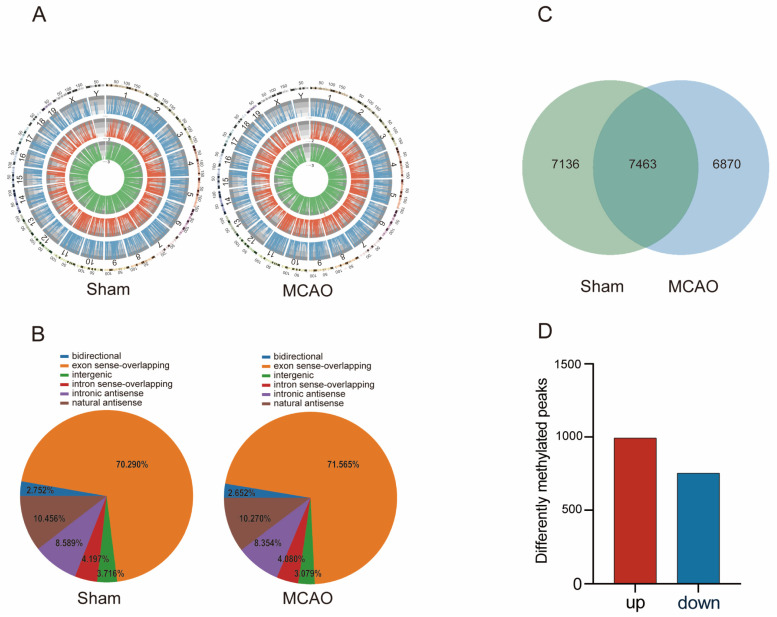
Overview of lncRNAs m^5^C in MCAO and normal mice. (**A**) Distribution of m^5^C at the chromosome level in MCAO and normal mice. (**B**) The source distribution of m^5^C-methylated lncRNAs. (**C**) Distribution of m^5^C peaks in MCAO and normal mice. (**D**) The number of up-regulated and down-regulated significantly changed m^5^C peaks in MCAO mice compared with the Sham group.

We conducted a comprehensive investigation into the distribution of lncRNAs m^5^C methylation sites across the genome in mice subjected to MCAO and in normal mice (Figure 3A). A total of 14,599 m^5^C peaks of lncRNAs in MCAO mice and 14,333 m^5^C peaks of lncRNAs in normal mice were identified. Our findings revealed that m^5^C methylation sites of lncRNAs were dispersed across all chromosomes. Furthermore, we observed that, in comparison to autosomal chromosomes, the distribution of lncRNAs m^5^C on sex chromosomes was reduced in both the MCAO and the Sham groups. Furthermore, a descriptive statistic was also made for the source of all m^5^C-methylated lncRNAs and observed that m^5^C methylation lncRNAs were mainly localized to the exon sense-overlapping regions, followed by natural antisense and intronic antisense (Figure 3B).

To gain further insights into the alterations following MCAO, we compared the distribution of lncRNAs m^5^C between the Sham and the MCAO group. Interestingly, the results showed that 7136 m^5^C peaks were unique to the MCAO group, while approximately 6870 m^5^C peaks were specific to normal mice (Figure 3C). Among the significantly changed m^5^C peaks (Figure 3D), a total of 992 m^5^C peaks showed upregulation, while 752 peaks exhibited down-regulation (*p* < 0.05, log_2_FC > 1). This includes lncRNA FosDT (transcript id: ENSMUST00000146541), whose increased expression leads to brain damage and neurological dysfunction following stroke. Compared to the control group, inhibiting FosDT can reduce the infarct volume and improve motor function recovery after ischemia, suggesting that FosDT may be a cytotoxic factor in rat hypoxic injury [44]. Table 1 presents the top 10 lncRNAs that exhibited differential methylation.

### 2.3. The Biological Information behind Differentially Methylated lncRNAs

**Figure 4:**
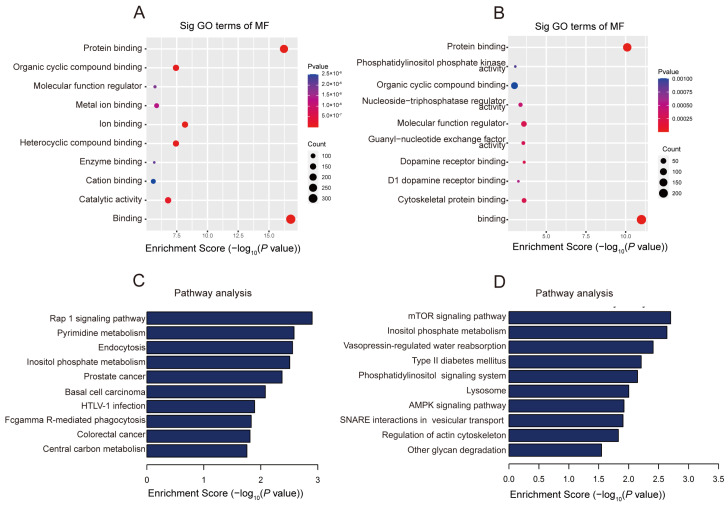
The biological information into lncRNAs m^5^C methylation in MCAO were unveiled through GO and KEGG analyses. (**A**) The top 10 gene ontology terms were significantly enriched for the hypermethylated lncRNAs. (**B**) The top 10 gene ontology terms were significantly enriched for the hypomethylated lncRNAs. (**C**) The 10 significantly enriched pathways for the hypermethylated lncRNAs. (**D**) The top 10 significantly enriched pathways for the hypomethylated lncRNAs.

In order to reveal the potential roles of differentially methylated lncRNAs, GO enrichment analysis was carried out. For the biological process (BP), the genes with upper methylation m^5^C locus were enriched in the cellular process and the primary metabolic process (Figure S1A), while the genes with hypomethylation m^5^C locus were enriched in cellular component organization or biogenesis and establishment of localization (Figure S1B). For cellular composition (CC), genes with up-regulated methylated m^5^C loci were enriched in an intracellular anatomical structure and a membrane-bounded organelle (Figure S1C). Genes with hypomethylation m^5^C loci were enriched in intracellular organelle and cytoplasm (Figure S1D). For molecular function (MF), the genes with hypermethylation m^5^C loci were mainly enriched in ion binding, metal ion binding and heterocyclic compound binding, and down-regulated methylation sites were related to dopamine receptor binding, phosphatidylinositol phosphate kinase activity, and nucleoside-triphosphatase regulator activity (Figure 4A,B). Dopamine receptors play a crucial role in the occurrence, development, and prognosis of stroke. Levodopa/benserazide treatment significantly improved the recovery of sensorimotor function after transient middle cerebral artery occlusion without affecting the infarct volume [45]. Metal ions play important roles in processes such as neuronal cell damage, oxidative stress, and inflammation. Iron has emerged as a key factor in post-reperfusion injury, and its metabolism in the brain is tightly regulated. Imbalances in iron metabolism, including overload and deficiency, have been shown to affect the outcome of ischemic stroke [46]. Copper acts as a catalytic cofactor in various physiological processes such as energy metabolism, mitochondrial respiration, and antioxidation. Low levels of copper ions can exacerbate tissue ischemia/reperfusion injury, while optimal levels of copper ions can improve tissue damage [47]. The oxidized purine-nucleoside triphosphatase (hMTH1) is a repair enzyme for oxidative DNA damage in brain tumors and neurons of Alzheimer’s disease. In the acute or subacute phase of cerebral infarction, astrocytes show a strong immunoreactivity to hMTH1, and oxidative stress rapidly induces the accumulation of hMTH1 [48].

The Kyoto Encyclopedia of Genes and Genomes (KEGG) was analyzed to identify path ways that may involve differentially methylated genes (Figure 4C,D). The results show that genes with up-regulated methylation in MCAO are mainly involved in the Rap1 signaling pathway, Pyrimidine metabolism pathways and Fcgamma R-mediate phagocyytosis. For genes with down-regulated methylation m^5^C, they are mainly involved in the mTOR signaling pathway, inositol phosphate pathways and the AMPK signaling pathway. MTOR is a serine/threonine protein kinase of 289 kDa. Studies have shown that activated mTOR/p70S6k kinase plays an important role in regulating protein synthesis and cell growth [49]. The expression of Fc receptors (FcRs) is believed to be associated with inflammatory cascades. In a mouse model of MCAO (middle cerebral artery occlusion), the mortality rate of FcgammaR knockout (FcgammaR-/-) mice is significantly reduced, possibly primarily attributed to the inhibition of activation and infiltration of inflammatory cells [50]. The activation of the sphingosine-1-phosphate/phosphatidylinositol-3-kinase/Akt pathway regulates a variety of cell events, including promoting cell proliferation, survival, migration, and inhibiting apoptosis, which may play a key role in neuroprotection [51]. AMPK has a protective effect on global cerebral ischemia. Recent studies have shown that the activation of the AMPK/Nrf2 pathway prevents ischemic stroke through its anti-inflammatory and antioxidant effects [52].

### 2.4. Gene Expression Profile following MCAO

**Figure 5:**
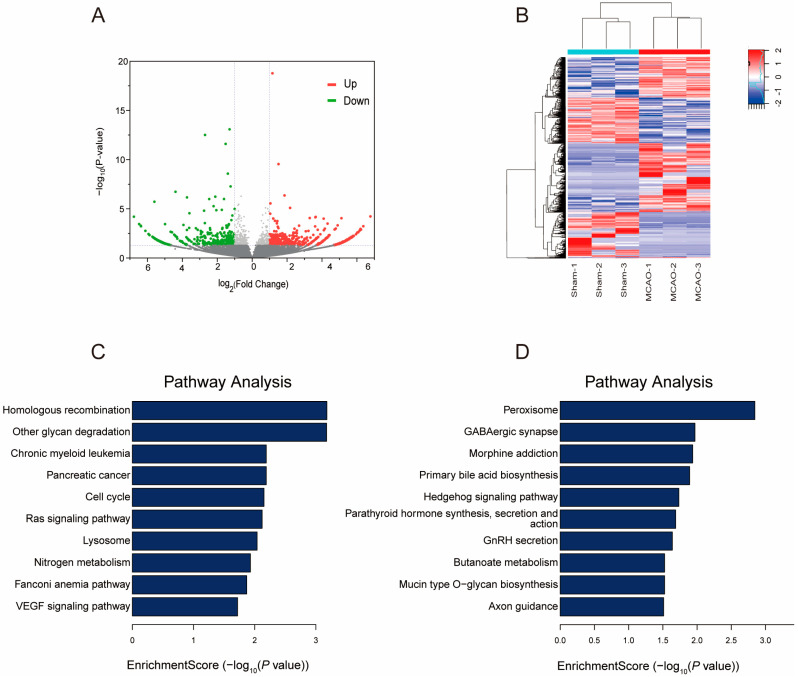
LncRNA expression changes after MCAO. (**A**) The volcano plot illustrates the lncRNAs that are significantly up-regulated and down-regulated following MCAO (fold changes ≥ 2 and *p* value < 0.05). (**B**) Cluster analysis of differently expressed lncRNAs. (**C**) The major significantly enriched pathways for the up-regulated genes. (**D**) The major significantly enriched pathways for the down-regulated genes.

To elucidate the association between gene expression and m^5^C methylation levels in greater detail, we measured the expression level of lncRNAs in mice cerebral cortex after MACO by RNA-seq. A total of 540 up-regulated lncRNAs and 445 down-regulated lncRNAs were identified (Figure 5A). The top 10 differently expressed lncRNAs were listed (Table 2). The results of cluster analysis showed that the expression levels of differently expressed lncRNAs could distinguish the MCAO group from the normal mouse group and it was relatively consistent within groups (Figure 5B).

We conducted KEGG analysis to identify pathways potentially involving differentially expressed genes (Figure 5C,D). The results revealed that up-regulated lncRNAs in MCAO were predominantly associated with the homologous recombination, RAS signaling pathway, and VEGF signaling pathway. Conversely, down-regulated lncRNAs were primarily linked to the peroxisome, GABAergic synapse, and morphine addiction. Gamma-aminobutyric acid (GABA) serves as the primary inhibitory neurotransmitter in the brain, and the modulation of cortical GABA activity plays a pivotal role in human motor learning. [53]. During cerebral ischemia, vascular endothelial growth factor-A (VEGF-A) secreted by astrocytes directly disrupted the blood-brain barrier [54]. However, the interleukin-9 can improve neurological functional deficit scores and reduce the infarct area in the ischemic brain tissue of middle cerebral artery occlusion rats by reducing astrocyte-derived factors such as VEGF-A and matrix metalloproteinase-9 (MMP-9) induced by oxygen-glucose deprivation (OGD) [55]. The Ras signaling pathway is involved in various processes such as cell survival, proliferation, and inflammation. Activation of the Ras-related C3 botulinum toxin substrate 1 (Rac1) signaling contributes to functional recovery in mice after ischemic stroke [56].

### 2.5. Combined Analysis of m^5^C Methylation and Gene Expression after MCAO

**Figure 6:**
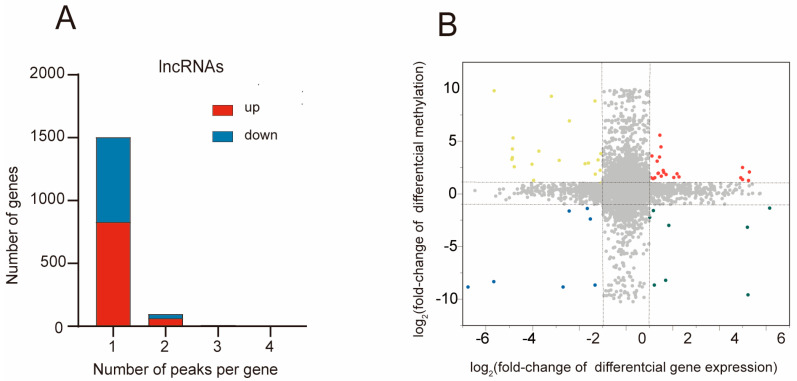
Combined examination of m^5^C methylation and lncRNA expression post-MCAO. (**A**) The distribution of altered m^5^C peaks per lncRNAs. (**B**) The four-quadrant graph illustrates the correlation between lncRNA m^5^C methylation and its corresponding lncRNA expression. Yellow (top-left quadrant): Methylation upregulation with gene expression downregulation. Red (top-right quadrant): Methylation and gene expression upregulation. Blue (bottom-left quadrant): Methylation and gene expression downregulation. Green (bottom-right quadrant): Methylation downregulation with gene expression upregulation.

To investigate the correlation between gene expression and m^5^C methylation, we performed a joint analysis with differently methylated and expressed lncRNAs. We firstly assessed the number of methylated m^5^C peaks with their corresponding lncRNAs. By analyzing the distribution of m^5^C modification peaks in each gene, we observed that the majority of genes (1504/1744) harbor one m^5^C methylation peak (Figure 6A). The methylated peaks that are up-regulated or down-regulated show a similar distribution across the genes (lncRNAs with one up-regulated m^5^C modification peak vs. lncRNAs with one down-regulated m^5^C modification peak: 83.77% vs. 89.36%; lncRNAs with two up-regulated m^5^C modification peaks vs. lncRNAs with two down-regulated m^5^C modification peaks: 6.4% vs. 4.5%).

Next, we aimed to explore the relationship between the differently methylated and expressed lncRNAs (Figure 6B), The integrated analysis of m^5^C methylation and lncRNA expression levels involved peaks with a log2 fold change > 1 and *p* < 0.05, along with lncRNAs exhibiting a log2 fold change > 1 and *p* < 0.05. Consequently, we identified 57 lncRNAs for which both their m^5^C peaks and lncRNA expression levels exhibited significant changes. Among these, 22 lncRNAs exhibited simultaneous up-regulation, while 7 lncRNAs showed concurrent down-regulation in their levels. Additionally, 8 genes exhibited up-regulated lncRNA expression coupled with down-regulated m^5^C peaks, and 20 genes displayed down-regulated lncRNA expression alongside up-regulated m^5^C peaks.

## 3. Discussion

The pivotal role of epigenetic changes in health and disease has been extensively established. Recent research has highlighted the significance of epigenetic modifications in the development and progression of various diseases, such as cancer, diabetes, chronic kidney disease, and neurodegenerative disorders [57,58]. Increasingly, studies are revealing the involvement of epigenetic modifications in neurobiology and nervous system diseases. One notable epigenetic modification, 5-methylcytosine (m^5^C), holds equal importance and plays a vital role in multiple biological processes. These processes encompass tRNA stabilization, rRNA assembly, functional stress responses, embryonic development, and organ development [59,60,61,62]. Alterations or mutations in RNA m^5^C methyltransferases, responsible for modifying m^5^C, have been associated with various pathological conditions, including neurological diseases and cancers [63]. Following transient middle cerebral artery occlusion (MCAO), levels of m^5^C increased in the cortex and striatum of mice [64]. Furthermore, isolated mouse brain mitochondria exhibited elevated m^5^C levels, as well as increased expression of DNMT1 and DNMT3A, at 72 h of reperfusion after MCAO [65]. The conversion of m^5^C to 5-hydroxymethylcytosine (5hmC) is catalyzed by ten-eleven translocation (Tet) enzymes, and the inhibition of Tet2 activity led to reduced 5hmC modification, associated with an augmented infarct volume following ischemic injury [66]. These findings suggest that alterations in m^5^C methylation patterns following a stroke may serve as potential biomarkers and therapeutic targets for the treatment of ischemic stroke.

We employed MeRIP-seq to sequence the m^5^C-modified lncRNAs peaks in mice subjected to MCAO as well as in normal mice. Our investigation revealed distinctive methylation peaks within lncRNAs, with significant variations in both the quantity and distribution of these peaks between the MCAO and normal mouse samples. The findings demonstrated markedly higher methylation frequencies and methylation levels of specific genes in the MCAO group as opposed to those in the normal mice. This compellingly established a clear association between m^5^C modifications and MCAO. After MCAO, we observed significant alterations in the expression levels and methylation status of certain genes, suggesting their potential as novel drug targets. The intricate mechanisms of these genes in stroke development warrant further exploration.

Subsequently, we explored the functional implications of these differential methylation patterns in lncRNAs. KEGG enrichment analysis uncovered that the genes under consideration regulated by lncRNAs exhibiting significant differential expression were primarily associated with peroxide and GABA pathways, as well as glycan degradation pathways. Furthermore, GO and KEGG enrichment analysis uncovered that differentially methylated lncRNAs were predominantly linked to mTOR signal transduction, Rap1 signal pathway transduction, pyrimidine metabolism, dopamine receptor binding, and phosphatidylinositol phosphate kinase activity.

Dopamine may help the recovery of motor function and programmed learning in stroke patients through neuronal plasticity and the regulation of cell proliferation and inflammation [67,68,69]. In MCAO, the activation of dopamine 1 (D1) and dopamine 2 (D2) receptors significantly increased the level of GDNF (glial cell line-derived neurotrophic factor) in the infarct core and peri-infarct area after tMCAO [70]. Phosphorylation of mTOR/p70S6k kinase has an anti-injury protective effect. Its activation can protect cells against ischemic damage in the central nervous system [71]. Studies have shown that GABA levels decrease 3 to 12 months after stroke. The changes in GABA in individual patients were significantly correlated with the improvement of motor performance after stroke [72]. The activation of Epac/Rap1 signal pathway has a neuroprotective effect on CI/R-damaged brain through the recovery of blood-brain barrier, while Rap1 inhibitor GGTI298 and Rac1 inhibitor NSC23766 inhibiting Epac pathway can destroy the blood-brain barrier and aggravate brain injury [73]. The results of this study indicate that these enriched signaling pathways are consistent with the pathological regulatory mechanism of stroke.

Our study has unveiled notable distinctions in both the quantity and distribution of m^5^C between the stroke group of mice MCAO model and the control group. These findings carry significant implications for advancing our comprehension of the regulatory mechanisms involving m^5^C in stroke. This study offers fresh insights into the potential discovery of markers for stroke diagnosis, progression, and novel targets for therapeutic interventions. However, it is important to underscore that further exploration is necessary to fully elucidate the epigenetic changes associated with stroke.

## 4. Methods

### 4.1. MCAO Models Construction

Male C57 mice of SPF grade (12–14 weeks) were fed in a 12 h light/dark environment one week before operation and were allowed to eat and drink freely. All animal experiments were performed in accordance with the guidelines for laboratory animal experiments established by the Animal Experimental Center and Ethics Committee of Zhongnan Hospital of Wuhan University (IACUC: ZN2022014). During the operation, 1.3% pentobarbital sodium was injected intraperitoneally to anesthetize mice. After making a median incision in the neck, the right common carotid artery, internal carotid artery and external carotid artery were exposed. The distal end of the external carotid artery was ligated with 6-0 silk thread at the 4 mm distance from the bifurcation of the common carotid artery. The common carotid artery was closed by an arterial clamp. A treated nylon thread was inserted along the common carotid artery to the middle cerebral artery. After ischemia for 60 min, we removed the thread bolt, ligated the proximal end of the external artery, sutured and sterilized the neck wound and put the mice on a heating pad, which was raised normally after waking up. Then, 24 h after operation, the anesthetized mice were injected intraperitoneally, and the brains were taken out for subsequent experiment.

### 4.2. TTC (2,3,5-Triphenyltetrazolium chloride) Staining

PBS was used to configure 1% TTC. The brain tissue sections were placed in 10 mL TTC solution for 10 min incubation at 37 °C. The brain slices were turned from time to time to make sure the tissue stained evenly. The normal brain tissue was bright red after staining, while the infarcted area was pale. The percentage of infarcted brain tissue was calculated by diving infarct volume by brain volume.

### 4.3. Evaluation of Neurological Deficit in Mice by Longa Biological Score

Longa biological score was used to evaluate the neurological deficits in mice. Specific scoring criteria: 1. No neurological deficits: 0 score; 2. The front paw of the paralyzed side cannot be fully extended: 1 score; 3. Turn to the paralyzed side while walking: 2 scores; 4. Fall on the paralyzed side while walking: 3 scores; 5. Cannot walk automatically, there is a loss of consciousness: 4 scores.

### 4.4. Nissl Staining

After successive dehydration in alcohols of different concentrations (75% alcohol for 4 h, 85% alcohol for 2 h, 90% alcohol for 1.5 h, 95% alcohol for 1 h, absolute ethanol for 1 h), the brain tissues from MCAO and control group mice were embedded in paraffin (60 °C). Paraffin blocks were then cut into thin slices with a thickness of 10 μm. Subsequently, the paraffin sections were sequentially placed in the following solutions: xylene I (10 min)—xylene II (10 min)–anhydrous ethanol I (5 min)—anhydrous ethanol II (5 min)—95% ethanol (3 min)—90% ethanol (3 min)—80% ethanol (2 min)—70% ethanol (2 min), followed by a 2 min wash with distilled water. The sections were then stained in a preheated 60 °C 1% cresyl violet staining solution for 40 min, then washed three times in distilled water and dehydrated in 95% ethanol. Finally, observation and image collections were performed using an Olympus BX53 biological microscope.

### 4.5. Immunofluorescence Staining

Brain specimens were dissected and preserved in 4% paraformaldehyde (24 h, 4 °C). The tissues were then dehydrated using a gradient of alcohol and subsequently embedded in paraffin. Sections were cut using a Leica RM 2016 rotary microtome (Leica Microsystems, Wetzlar, Germany). The sections were stored in a −80 °C freezer until analysis. Brain tissue sections were placed in a container with antigen retrieval buffer (0.01 M citrate buffer, pH 6.0) and heated for 15 min to perform antigen retrieval. The samples were washed three times with PBS (pH 7.4), 3 min each time, and then incubated with goat serum for 30 min at room temperature to reduce non-specific staining. Next, the primary antibody was added and incubated overnight (15 h) at 4 °C in a humidified chamber. Subsequently, the sections were washed three times with PBST (PBS with Tween-20), 3 min each time, and then incubated with the secondary antibody at 37 °C in a humidified chamber for 1 h. Sections were incubated with a proteinase K solution at room temperature for 20 min and then subjected to apoptosis staining using a cell apoptosis detection kit (Vazyme, A113-03). DAPI (Beyotime, C1002) was used for nuclear counterstaining. Fluorescent images were captured using the Olympus BX53 biological microscope.

### 4.6. Extraction of RNA and Preparation of RNA-Seq Library

Fresh brain tissues were immediately collected and stored in liquid nitrogen. Total RNA of brain tissues was extracted by Trizol reagent. The ribosomal RNA (rRNA) was removed from the total RNA by the GenSeq^®^ rRNA Removal Kit (Cloud-Seq Biotechnology, Shanghai, China). According to the instructions provided by the manufacturer, the RNA-seq library was constructed by GenSeq^®^ Low Input RNA Library Prep Kit (GenSeq, Inc.). The RNA-seq library was qualitatively controlled and quantified by BioAnalyzer 2100 system (Agilent Technologies, Santa Clara, CA, USA). Then the Illumina NovaSeq 6000 instrument was used for 150 bp double-terminal sequencing.

### 4.7. Preparation of Methylated RNA Immunoprecipitation Sequencing (MeRIP-Seq) Library

The purified RNA was fragmented at 70 °C for 5–7 min in a 20 μL buffer (18 μL total RNA and 2 μL 10× Fragmentation Buffer). After the fragmented reaction, 2 µL of Stop Buffer was added and mixed. Subsequently, RNA fragment sizes and concentrations were assessed using the Agilent Bioanalyzer and Agilent RNA 6000 Pico kit. Fragmented RNA size distribution was confirmed by agarose gel electrophoresis (RNA fragment size approximately ~200 nt). The m^5^C antibody was incubated with PGM magnetic beads at room temperature for 1 h to facilitate binding. The fragmented RNA, nuclease-free water, and 5× IP buffer were combined to create a 250 μL MeRIP reaction mixture, which was then incubated with immunoprecipitation magnetic beads for 4 h. Then, the complex was washed several times with RLT Buffer to elute RNA from the complex. The MeRIP-Seq library was prepared using the GenSeq^®^ Low Input Whole RNA Library Prep Kit (GenSeq, Inc.). The constructed libraries underwent quality control using an Agilent 2100 Bioanalyzer and were subsequently subjected to high-throughput sequencing on an Illumina NovaSeq sequencer. 

### 4.8. Data Processing and Bioinformatics Analysis

The sequencing was performed using the Illumina NovaSeq 6000 sequencer, generating raw data. Quality control was conducted using Q30 scores, and the cutadapt software (v1.9.3) was employed to trim adapters and remove low-quality reads, resulting in high-quality clean reads.

For the MeRIP-Seq library, the clean reads from the input library were aligned to the mouse reference genome (UCSC MM10) using STAR software (v2.5.1b) and Hisat2 software (v2.0.4). Subsequently, the MACS software (MACS 1.4.2) was employed to identify methylated peaks in each sample, and diffReps software (diffreps 1.55.6) was used to detect differentially methylated genes. A custom program was applied to filter peaks located on exons of mRNA, lncRNA, and circRNA and annotate them accordingly.

For the RNA-seq library, the high-quality reads were aligned to the reference genome using hisat2 software (v2.0.4). HTSeq software (v0.9.1) was then used to obtain transcript-level raw count data for lncRNA expression profiles. The edgeR software (v3.16.5) was employed to normalize the data and calculate fold changes and P-values between the two sample groups, enabling the identification of differentially expressed lncRNAs. The differentially methylated and expressed lncRNAs related protein-coding genes were enriched and analyzed. Functional enrichment analysis and pathway enrichment analysis by Gene Ontology (GO) and Kyoto Encyclopedia of Genes and Genomes (KEGG) were conducted to annotate and predict the possible functions of these lncRNAs in stroke.

## Figures and Tables

**Table 1:** Top 10 differently m^5^C-methylated lncRNAs in the MCAO group compared to the Sham group.

LncRNA	Chromosome	Peak Start	Peak End	*p*-Value	Log_2_ (Fold Change)	Methylated Regulation
* ENSMUST00000176434 *	chr17	24,516,301	24,516,599	3 × 10^−9^	9.789697	up
* ENSMUST00000133764 *	chr4	42,164,721	42,164,736	3.66 × 10^−9^	9.843136	up
* ENSMUST00000130394 *	chr11	69,601,781	69,602,080	8.93 × 10^−9^	9.890872	up
* ENSMUST00000123459 *	chr11	3,533,741	3,534,000	3.44 × 10^−9^	9.960581	up
* ENSMUST00000004377 *	chr6	124,732,221	124,732,440	8.64 × 10^−9^	9.964196	up
* ENSMUST00000089622 *	chr6	83,441,761	83,442,105	3.47 × 10^−9^	−10.6774	down
* ENSMUST00000141371 *	chr3	88,542,675	88,542,781	4.29 × 10^−9^	−10.5481	down
* ENSMUST00000069298 *	chr19	42,779,821	42,780,060	3.78 × 10^−9^	−10.5284	down
* ENSMUST00000184280 *	chr7	16,825,789	16,825,850	1.05 × 10^−8^	−10.3767	down
* ENSMUST00000149651 *	chr6	119,215,341	119,215,660	1.33 × 10^−8^	−10.2427	down

**Table 2:** Top 10 differently expressed lncRNAs in the MCAO group compared with the Sham group.

LncRNA	Chromosome	Gene Start	Gene End	Log_2_ (Fold Change)	*p*-Value	Regulation
*ENSMUST00000127704*	chr3	116,326,038	116,424,032	6.7759264	5.964 × 10^−9^	up
*ENSMUST00000159482*	chr6	29,473,161	29,484,144	6.3668936	0.000591	up
*AK076596*	chr14	95,882,943	95,884,433	6.2356941	0.0013011	up
*ENSMUST00000117051*	chr2	175,038,120	175,041,036	6.2093761	0.0011026	up
*ENSMUST00000121563*	chr5	88,739,246	88,739,729	6.1554138	0.0016276	up
*ENSMUST00000140938*	chr1	10,595,365	10,719,905	−6.771754	6.317 × 10^−9^	down
*ENSMUST00000134513*	chr2	157,561,008	157,561,855	−6.472668	0.0003559	down
*NR_029577*	chr9	108,568,318	108,568,392	−6.38121	0.0005161	down
*ENSMUST00000182466*	chr19	24,692,399	24,693,370	−6.367063	0.0005963	down
*AK041456*	chr6	31,795,668	31,798,497	−6.11473	0.001745	down

## Data Availability

Data is contained within the article and Supplementary Material.

## Supplementary Materials

The following supporting information can be downloaded at: https://www.mdpi.com/article/10.3390/ph17030384/s1, Figure S1: BP and CC analysis of differentially methylated lncRNAs; Table S1: Differential Expression lncRNAs Summary; Table S2: Differential Methylation lncRNAs Summary.
