# Supplementary material for: Transcriptome-Wide 5-Methylcytosine Profiling of lncRNAs in the Mouse Cerebral Ischemia Model
Source: Pharmaceuticals (Basel). 2024 Mar 18;17(3):384. doi: 10.3390/ph17030384 (PMC10974612; doi:10.3390/ph17030384)
Supplement: Supplementary file 1 [file pharmaceuticals-17-00384-s001.zip › Figure notes in Figure S1.pdf]

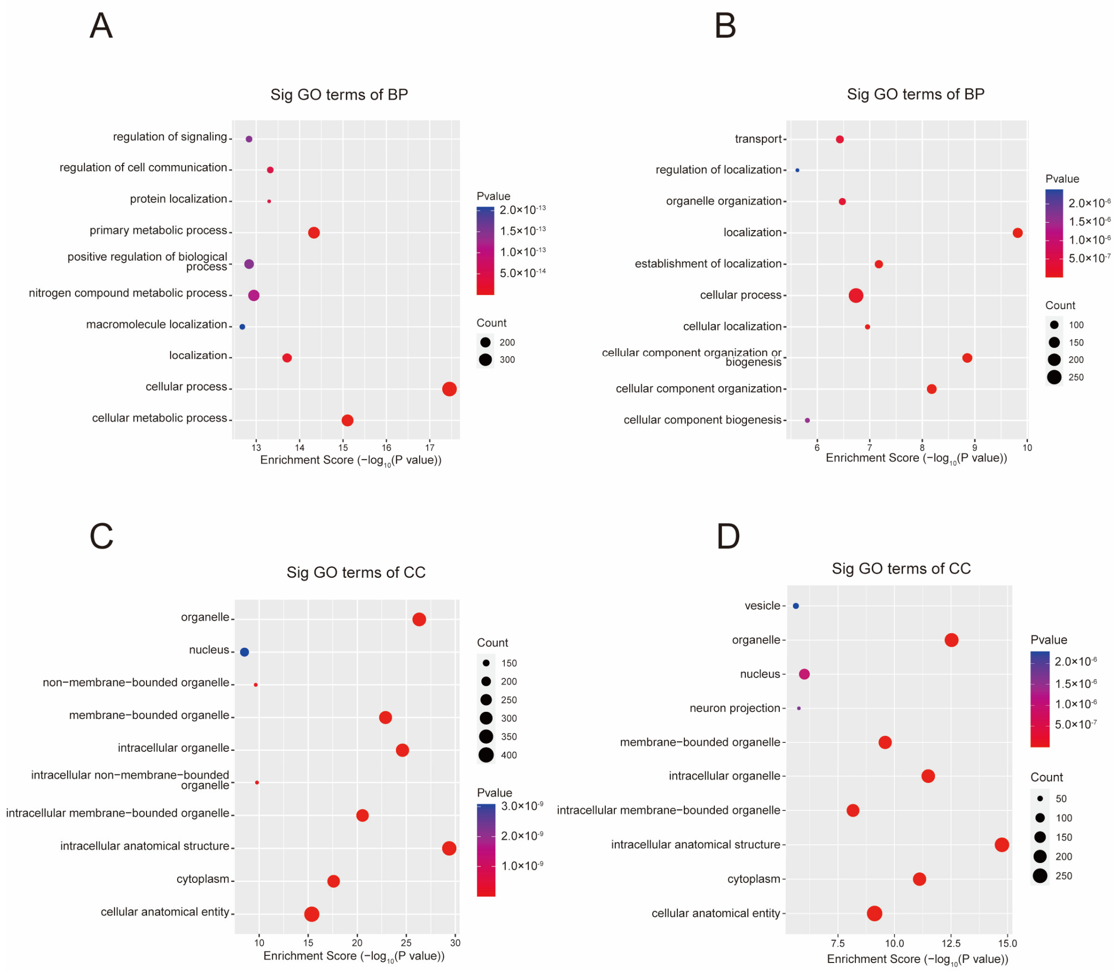

Figure S1. BP and CC analysis of differentially methylated lncRNAs. (A): Enriched biological processes (BP) of up-regulated differentially methylated lncRNAs. (B): Enriched biological processes (BP) of down-regulated differentially methylated lncRNAs. (C): cell component (CC) enriched of up-regulated differentially methylated lncRNAs. (D): cell component (CC) enriched of down-regulated differentially methylated lncRNAs.
